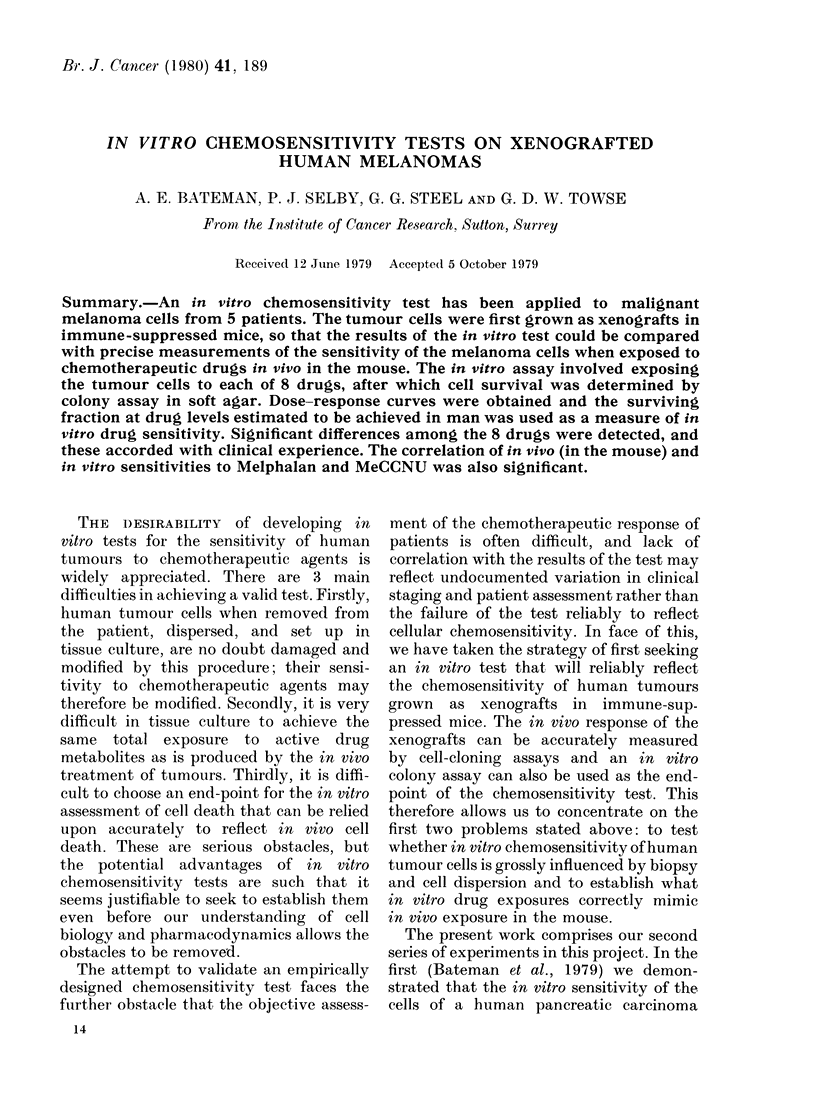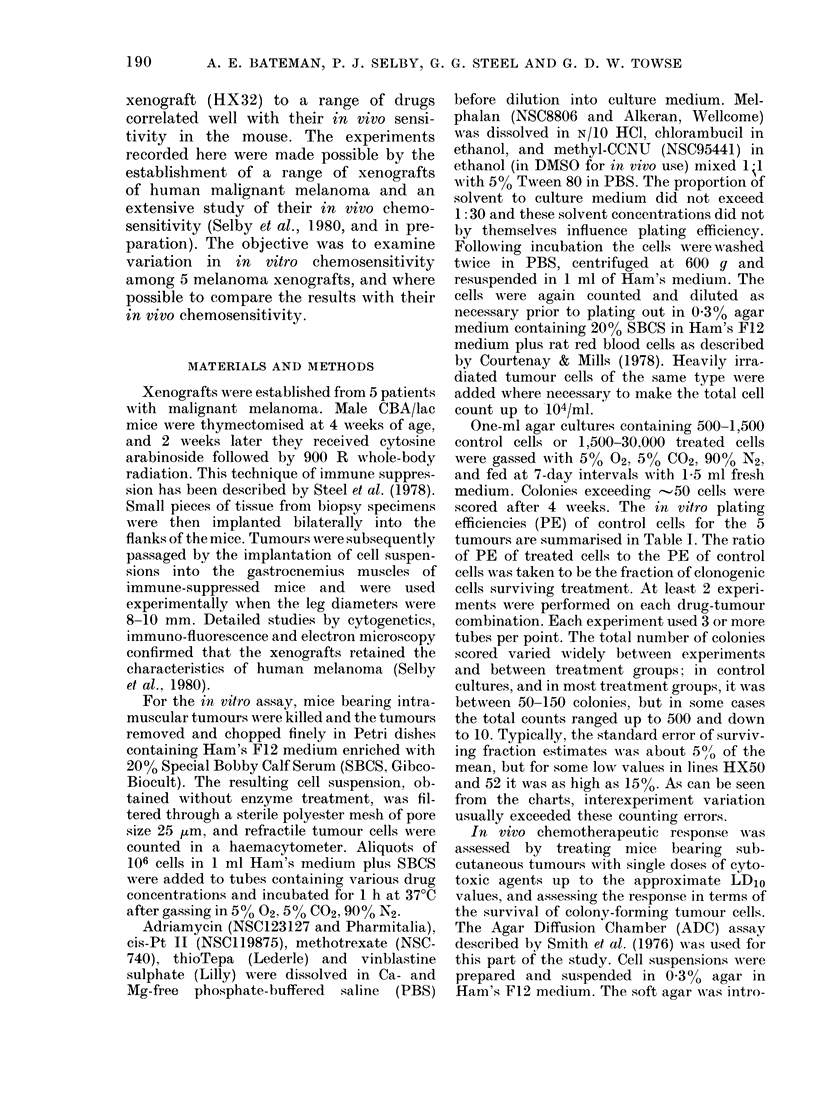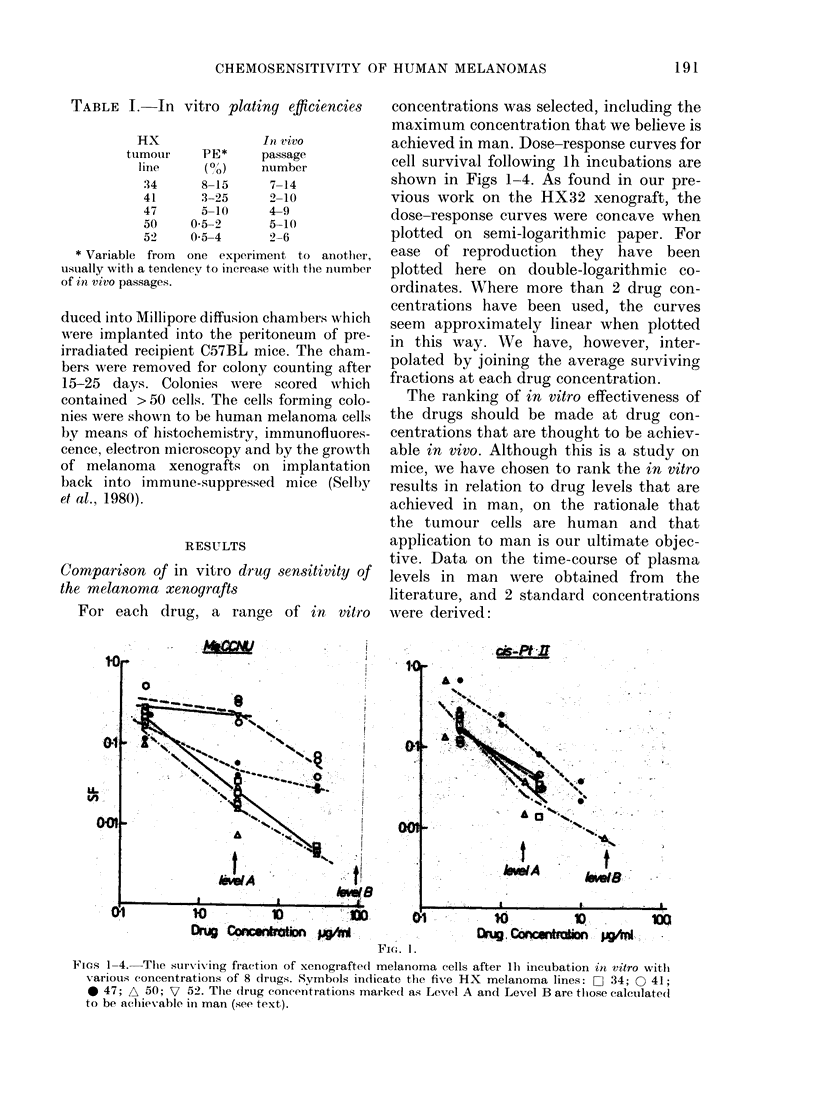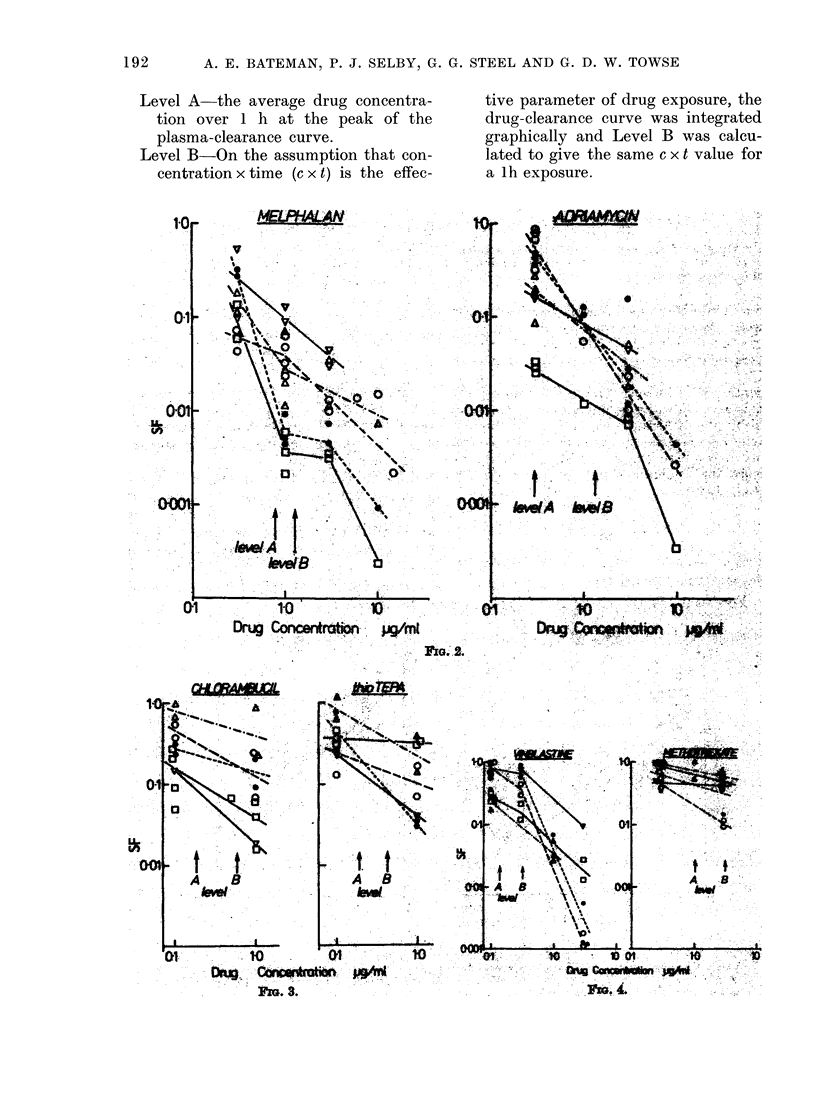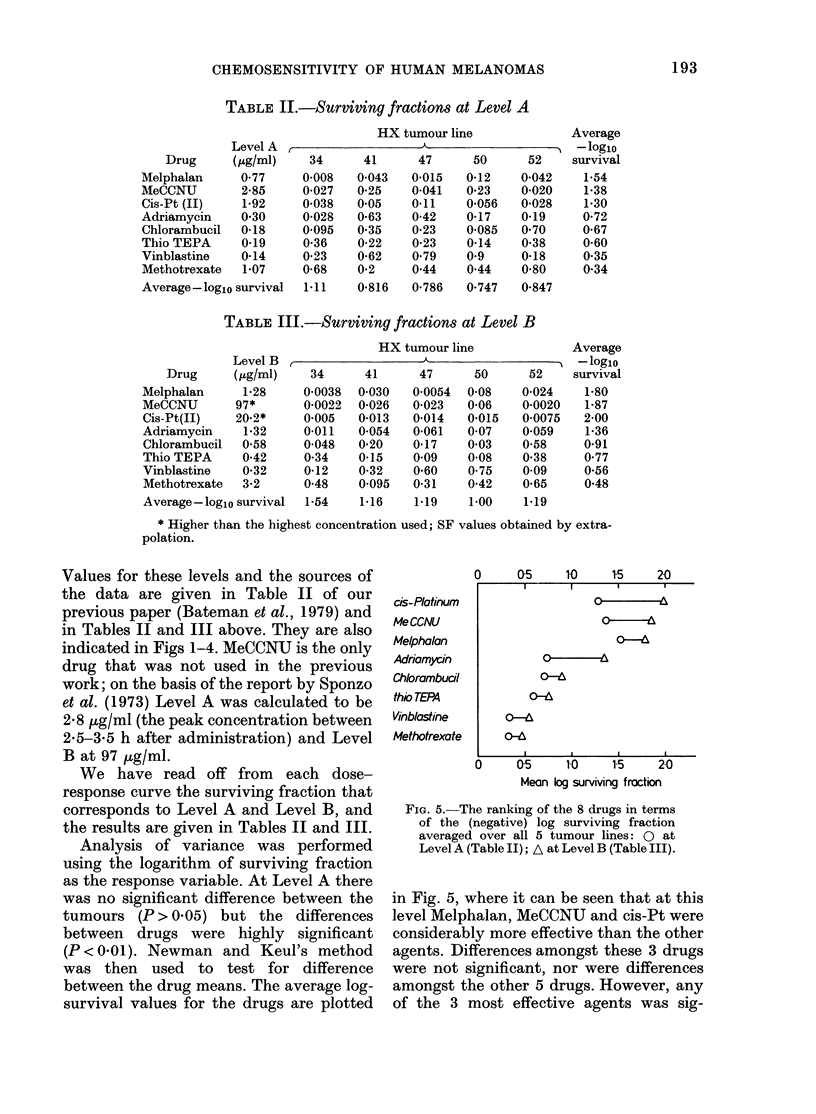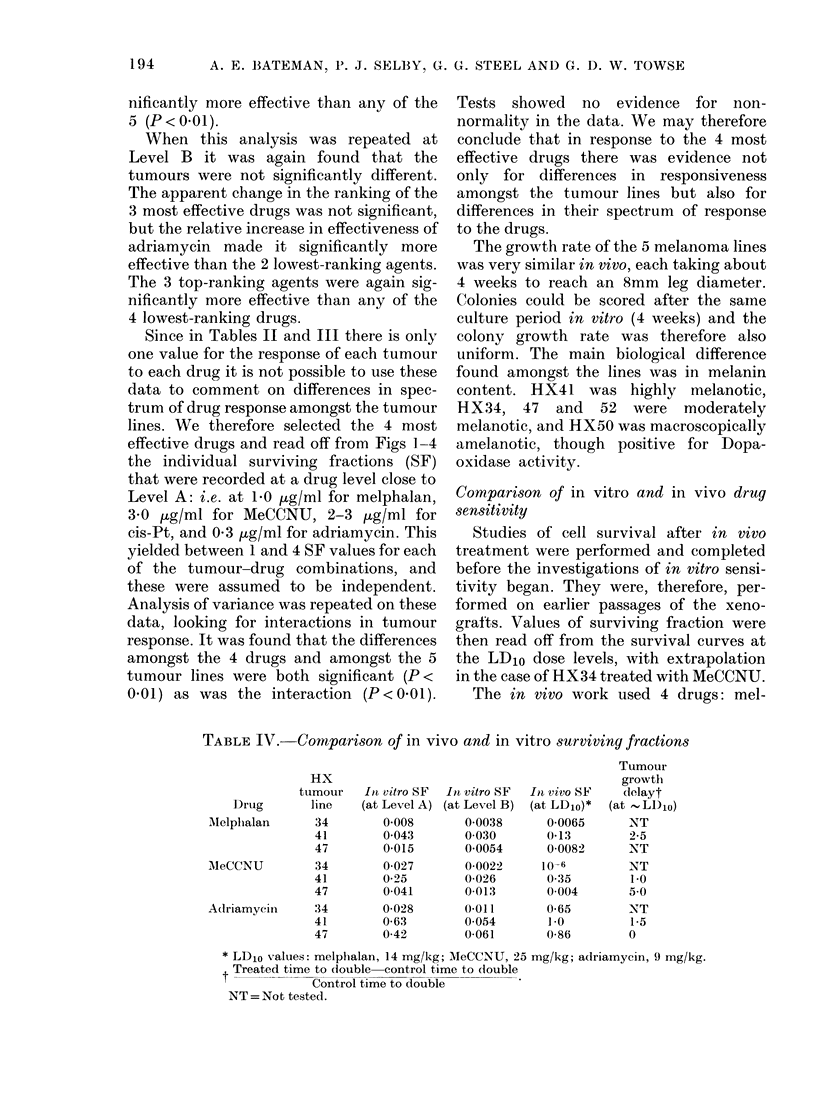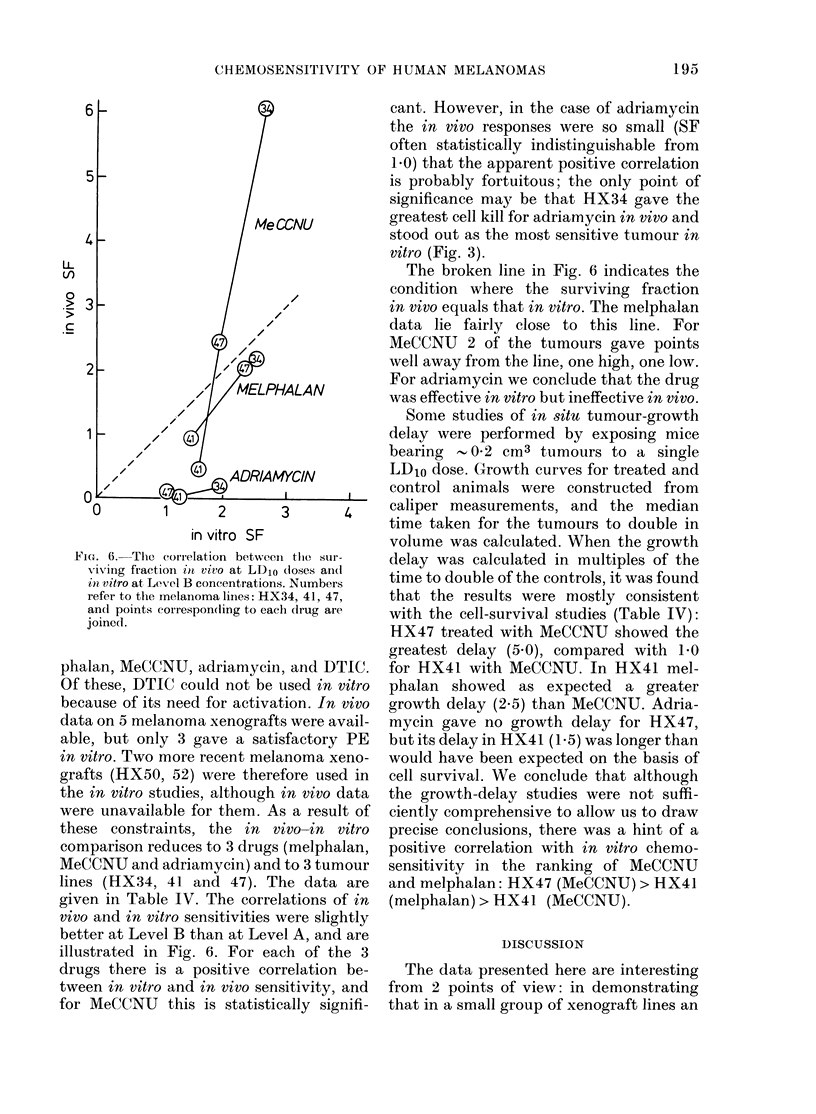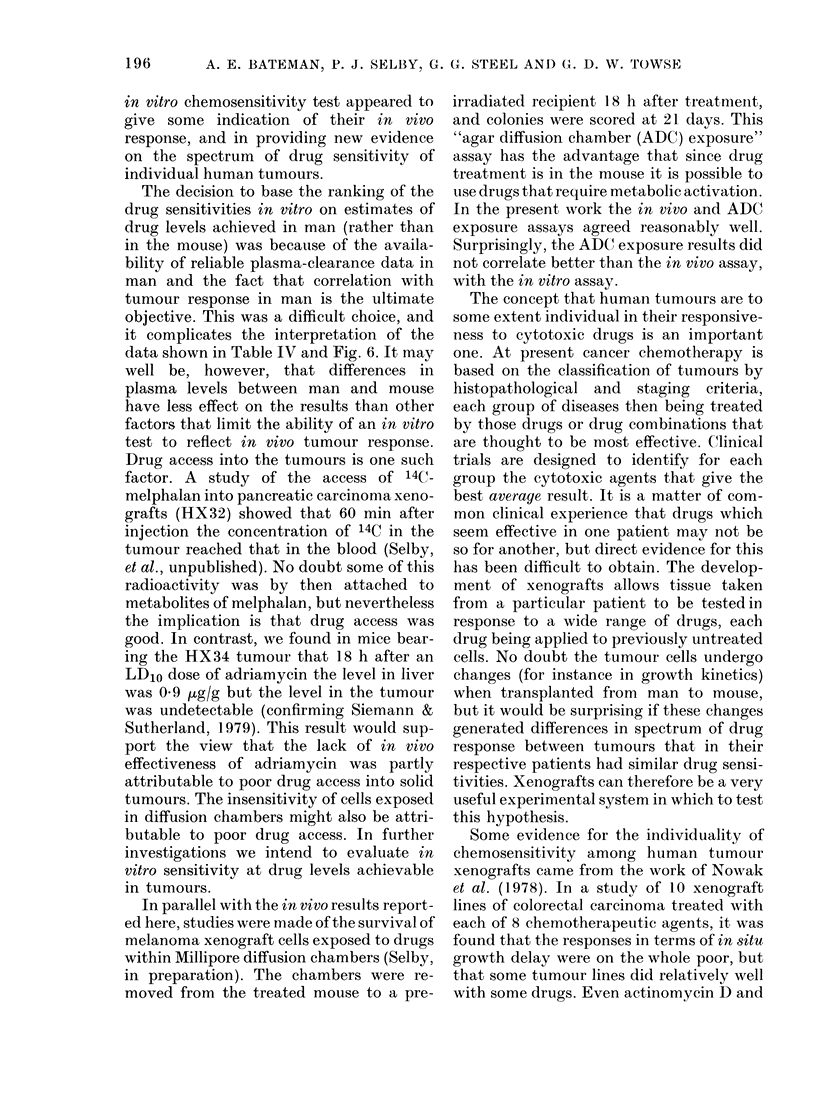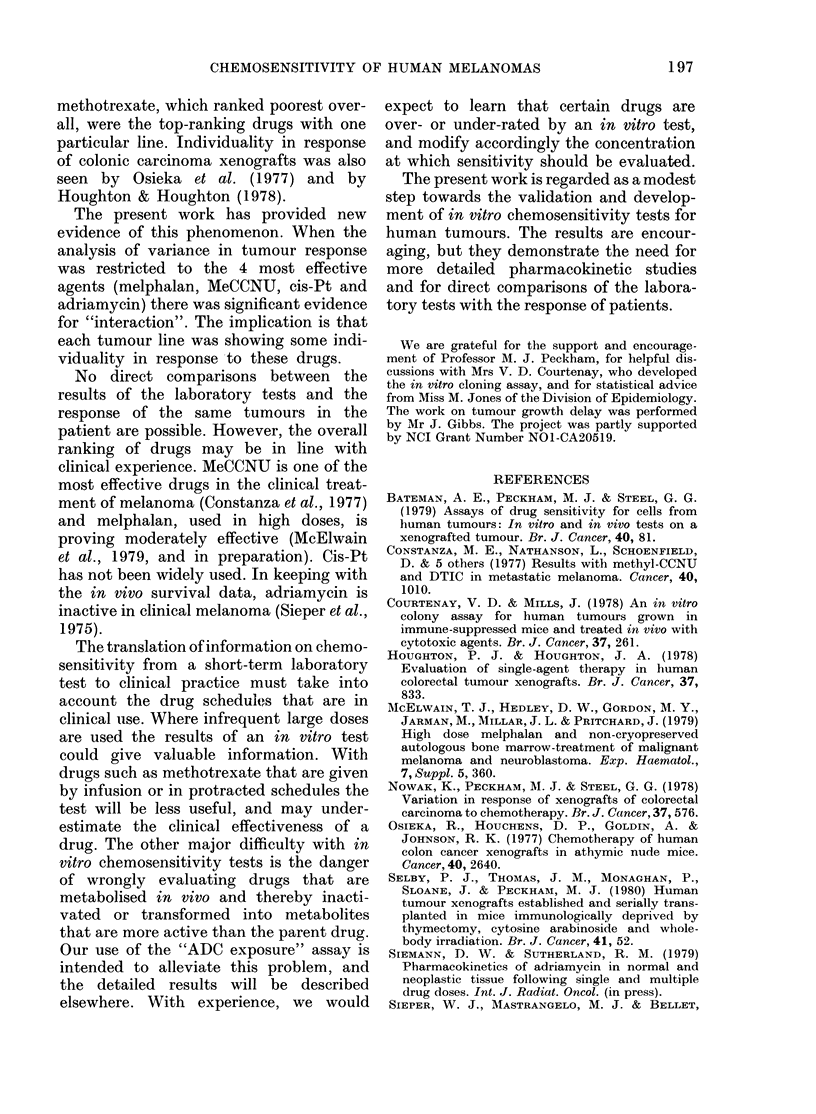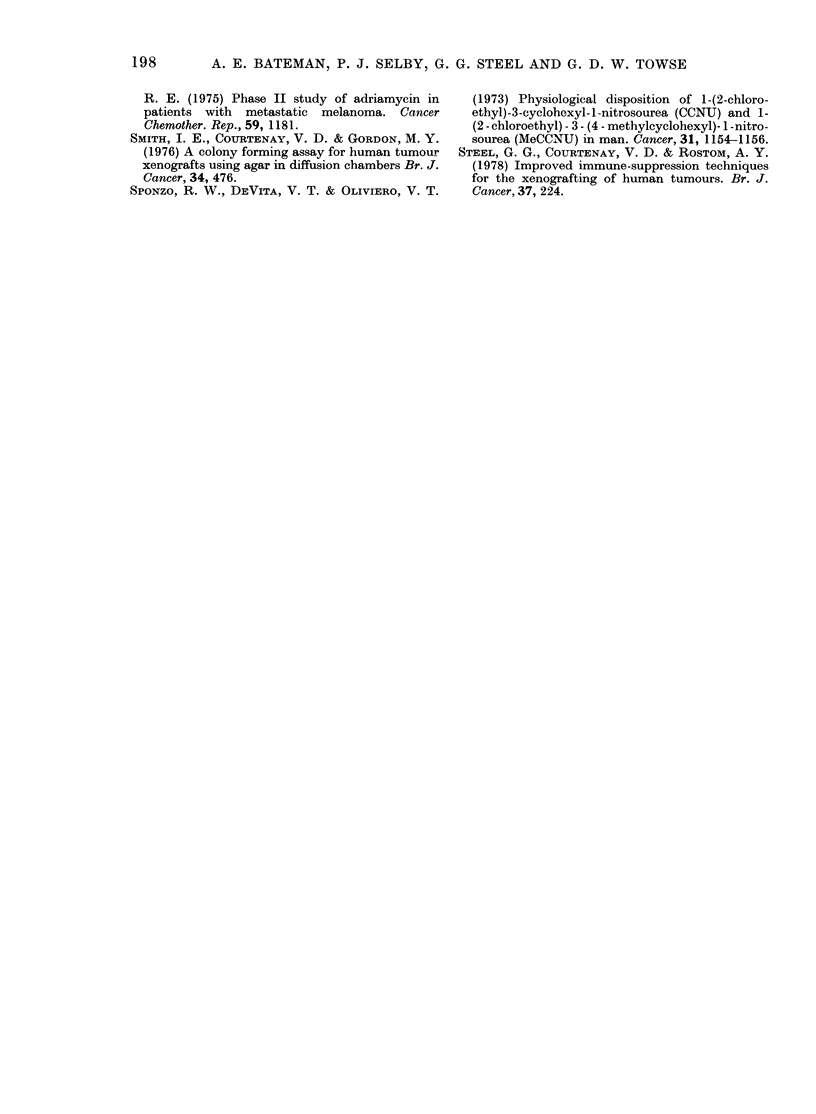# In vitro chemosensitivity tests on xenografted human melanomas.

**DOI:** 10.1038/bjc.1980.29

**Published:** 1980-02

**Authors:** A. E. Bateman, P. J. Selby, G. G. Steel, G. D. Towse

## Abstract

An in vitro chemosensitivity test has been applied to malignant melanoma cells from 5 patients. The tumour cells were first grown as xenografts in immune-suppressed mice, so that the results of the in vitro test could be compared with precise measurements of the sensitivity of the melanoma cells when exposed to chemotherapeutic drugs in vivo in the mouse. The in vitro assay involved exposing the tumour cells to each of 8 drugs, after which cell survival was determined by colony assay in soft agar. Dose-response curves were obtained and the surviving fraction at drug levels estimated to be achieved in man was used as a measure of in vitro drug sensitivity. Significant differences among the 8 drugs were detected, and these accorded with clinical experience. The correlation of in vivo (in the mouse) and in vitro sensitivities to Melphalan and MeCCNU was also significant.


*Br. J. Cancer* (1980) **41**, 189

### *IN VITRO* CHEMOSENSITIVITY TESTS ON XENOGRAFTED HUMAN MELANOMAS

A. E. BATEMAN, P. J. SELBY, G. G. STEEL AND G. D. W. TOWSE

*From the Institute of Cancer Research, Sutton, Surrey*

Received 12 June 1979 Accepted 5 October 1979

**Summary.—An *in vitro* chemosensitivity test has been applied to malignant melanoma cells from 5 patients. The tumour cells were first grown as xenografts in immune-suppressed mice, so that the results of the *in vitro* test could be compared with precise measurements of the sensitivity of the melanoma cells when exposed to chemotherapeutic drugs *in vivo* in the mouse. The *in vitro* assay involved exposing the tumour cells to each of 8 drugs, after which cell survival was determined by colony assay in soft agar. Dose–response curves were obtained and the surviving fraction at drug levels estimated to be achieved in man was used as a measure of *in vitro* drug sensitivity. Significant differences among the 8 drugs were detected, and these accorded with clinical experience. The correlation of *in vivo* (in the mouse) and *in vitro* sensitivities to Melphalan and MeCCNU was also significant.**

THE DESIRABILITY of developing *in vitro* tests for the sensitivity of human tumours to chemotherapeutic agents is widely appreciated. There are 3 main difficulties in achieving a valid test. Firstly, human tumour cells when removed from the patient, dispersed, and set up in tissue culture, are no doubt damaged and modified by this procedure; their sensitivity to chemotherapeutic agents may therefore be modified. Secondly, it is very difficult in tissue culture to achieve the same total exposure to active drug metabolites as is produced by the *in vivo* treatment of tumours. Thirdly, it is difficult to choose an end-point for the *in vitro* assessment of cell death that can be relied upon accurately to reflect *in vivo* cell death. These are serious obstacles, but the potential advantages of *in vitro* chemosensitivity tests are such that it seems justifiable to seek to establish them even before our understanding of cell biology and pharmacodynamics allows the obstacles to be removed.

The attempt to validate an empirically designed chemosensitivity test faces the further obstacle that the objective assessment of the chemotherapeutic response of patients is often difficult, and lack of correlation with the results of the test may reflect undocumented variation in clinical staging and patient assessment rather than the failure of the test reliably to reflect cellular chemosensitivity. In face of this, we have taken the strategy of first seeking an *in vitro* test that will reliably reflect the chemosensitivity of human tumours grown as xenografts in immune-suppressed mice. The *in vivo* response of the xenografts can be accurately measured by cell-cloning assays and an *in vitro* colony assay can also be used as the end-point of the chemosensitivity test. This therefore allows us to concentrate on the first two problems stated above: to test whether *in vitro* chemosensitivity of human tumour cells is grossly influenced by biopsy and cell dispersion and to establish what *in vitro* drug exposures correctly mimic *in vivo* exposure in the mouse.

The present work comprises our second series of experiments in this project. In the first (Bateman *et al.*, 1979) we demonstrated that the *in vitro* sensitivity of the cells of a human pancreatic carcinoma xenograft (HX32) to a range of drugs correlated well with their *in vivo* sensitivity in the mouse. The experiments recorded here were made possible by the establishment of a range of xenografts of human malignant melanoma and an extensive study of their *in vivo* chemosensitivity (Selby *et al.*, 1980, and in preparation). The objective was to examine variation in *in vitro* chemosensitivity among 5 melanoma xenografts, and where possible to compare the results with their *in vivo* chemosensitivity.

### MATERIALS AND METHODS

Xenografts were established from 5 patients with malignant melanoma. Male CBA/lac mice were thymectomised at 4 weeks of age, and 2 weeks later they received cytosine arabinoside followed by 900 R whole-body radiation. This technique of immune suppression has been described by Steel *et al.* (1978). Small pieces of tissue from biopsy specimens were then implanted bilaterally into the flanks of the mice. Tumours were subsequently passaged by the implantation of cell suspensions into the gastrocnemius muscles of immune-suppressed mice and were used experimentally when the leg diameters were 8–10 mm. Detailed studies by cytogenetics, immuno-fluorescence and electron microscopy confirmed that the xenografts retained the characteristics of human melanoma (Selby *et al.*, 1980).

For the *in vitro* assay, mice bearing intramuscular tumours were killed and the tumours removed and chopped finely in Petri dishes containing Ham's F12 medium enriched with 20% Special Bobby Calf Serum (SBCS, Gibco-Biocult). The resulting cell suspension, obtained without enzyme treatment, was filtered through a sterile polyester mesh of pore size 25 $\mu$m, and refractile tumour cells were counted in a haemacytometer. Aliquots of $10^6$ cells in 1 ml Ham's medium plus SBCS were added to tubes containing various drug concentrations and incubated for 1 h at 37°C after gassing in 5% $O_2$, 5% $CO_2$, 90% $N_2$.

Adriamycin (NSC123127 and Pharmitalia), cis-Pt II (NSC119875), methotrexate (NSC-740), thioTepa (Lederle) and vinblastine sulphate (Lilly) were dissolved in Ca- and Mg-free phosphate-buffered saline (PBS) before dilution into culture medium. Melphalan (NSC8806 and Alkeran, Wellcome) was dissolved in N/10 HCl, chlorambucil in ethanol, and methyl-CCNU (NSC95441) in ethanol (in DMSO for *in vivo* use) mixed 1:1 with 5% Tween 80 in PBS. The proportion of solvent to culture medium did not exceed 1:30 and these solvent concentrations did not by themselves influence plating efficiency. Following incubation the cells were washed twice in PBS, centrifuged at 600 *g* and resuspended in 1 ml of Ham's medium. The cells were again counted and diluted as necessary prior to plating out in 0·3% agar medium containing 20% SBCS in Ham's F12 medium plus rat red blood cells as described by Courtenay & Mills (1978). Heavily irradiated tumour cells of the same type were added where necessary to make the total cell count up to $10^4$/ml.

One-ml agar cultures containing 500–1,500 control cells or 1,500–30,000 treated cells were gassed with 5% $O_2$, 5% $CO_2$, 90% $N_2$, and fed at 7-day intervals with 1·5 ml fresh medium. Colonies exceeding ~50 cells were scored after 4 weeks. The *in vitro* plating efficiencies (PE) of control cells for the 5 tumours are summarised in Table I. The ratio of PE of treated cells to the PE of control cells was taken to be the fraction of clonogenic cells surviving treatment. At least 2 experiments were performed on each drug-tumour combination. Each experiment used 3 or more tubes per point. The total number of colonies scored varied widely between experiments and between treatment groups; in control cultures, and in most treatment groups, it was between 50–150 colonies, but in some cases the total counts ranged up to 500 and down to 10. Typically, the standard error of surviving fraction estimates was about 5% of the mean, but for some low values in lines HX50 and 52 it was as high as 15%. As can be seen from the charts, interexperiment variation usually exceeded these counting errors.

*In vivo* chemotherapeutic response was assessed by treating mice bearing subcutaneous tumours with single doses of cytotoxic agents up to the approximate $LD_{10}$ values, and assessing the response in terms of the survival of colony-forming tumour cells. The Agar Diffusion Chamber (ADC) assay described by Smith *et al.* (1976) was used for this part of the study. Cell suspensions were prepared and suspended in 0·3% agar in Ham's F12 medium. The soft agar was intro-

TABLE I.—In vitro *plating efficiencies*

| HX tumour line | PE* (%) | *In vivo* passage number |
|---|---|---|
| 34 | 8–15 | 7–14 |
| 41 | 3–25 | 2–10 |
| 47 | 5–10 | 4–9 |
| 50 | 0·5–2 | 5–10 |
| 52 | 0·5–4 | 2–6 |

* Variable from one experiment to another, usually with a tendency to increase with the number of *in vivo* passages.

duced into Millipore diffusion chambers which were implanted into the peritoneum of pre-irradiated recipient C57BL mice. The chambers were removed for colony counting after 15–25 days. Colonies were scored which contained >50 cells. The cells forming colonies were shown to be human melanoma cells by means of histochemistry, immunofluorescence, electron microscopy and by the growth of melanoma xenografts on implantation back into immune-suppressed mice (Selby *et al.*, 1980).

### RESULTS

### *Comparison of* in vitro *drug sensitivity of the melanoma xenografts*

For each drug, a range of *in vitro* concentrations was selected, including the maximum concentration that we believe is achieved in man. Dose–response curves for cell survival following 1h incubations are shown in Figs 1–4. As found in our previous work on the HX32 xenograft, the dose–response curves were concave when plotted on semi-logarithmic paper. For ease of reproduction they have been plotted here on double-logarithmic co-ordinates. Where more than 2 drug concentrations have been used, the curves seem approximately linear when plotted in this way. We have, however, interpolated by joining the average surviving fractions at each drug concentration.

The ranking of *in vitro* effectiveness of the drugs should be made at drug concentrations that are thought to be achievable *in vivo*. Although this is a study on mice, we have chosen to rank the *in vitro* results in relation to drug levels that are achieved in man, on the rationale that the tumour cells are human and that application to man is our ultimate objective. Data on the time-course of plasma levels in man were obtained from the literature, and 2 standard concentrations were derived:

FIG. 1.

FIGS 1–4.—The surviving fraction of xenografted melanoma cells after 1h incubation *in vitro* with various concentrations of 8 drugs. Symbols indicate the five HX melanoma lines: □ 34; ○ 41; ● 47; △ 50; ▽ 52. The drug concentrations marked as Level A and Level B are those calculated to be achievable in man (see text).

Level A—the average drug concentration over 1 h at the peak of the plasma-clearance curve.

Level B—On the assumption that concentration × time ($c \times t$) is the effective parameter of drug exposure, the drug-clearance curve was integrated graphically and Level B was calculated to give the same $c \times t$ value for a 1h exposure.

FIG. 2.

FIG. 3.

FIG. 4.

TABLE II.—*Surviving fractions at Level A*

| Drug | Level A (μg/ml) | HX tumour line 34 | 41 | 47 | 50 | 52 | Average $-\log_{10}$ survival |
|---|---|---|---|---|---|---|---|
| Melphalan | 0·77 | 0·008 | 0·043 | 0·015 | 0·12 | 0·042 | 1·54 |
| MeCCNU | 2·85 | 0·027 | 0·25 | 0·041 | 0·23 | 0·020 | 1·38 |
| Cis-Pt (II) | 1·92 | 0·038 | 0·05 | 0·11 | 0·056 | 0·028 | 1·30 |
| Adriamycin | 0·30 | 0·028 | 0·63 | 0·42 | 0·17 | 0·19 | 0·72 |
| Chlorambucil | 0·18 | 0·095 | 0·35 | 0·23 | 0·085 | 0·70 | 0·67 |
| Thio TEPA | 0·19 | 0·36 | 0·22 | 0·23 | 0·14 | 0·38 | 0·60 |
| Vinblastine | 0·14 | 0·23 | 0·62 | 0·79 | 0·9 | 0·18 | 0·35 |
| Methotrexate | 1·07 | 0·68 | 0·2 | 0·44 | 0·44 | 0·80 | 0·34 |
| Average $-\log_{10}$ survival | | 1·11 | 0·816 | 0·786 | 0·747 | 0·847 | |

TABLE III.—*Surviving fractions at Level B*

| Drug | Level B (μg/ml) | HX tumour line 34 | 41 | 47 | 50 | 52 | Average $-\log_{10}$ survival |
|---|---|---|---|---|---|---|---|
| Melphalan | 1·28 | 0·0038 | 0·030 | 0·0054 | 0·08 | 0·024 | 1·80 |
| MeCCNU | 97* | 0·0022 | 0·026 | 0·023 | 0·06 | 0·0020 | 1·87 |
| Cis-Pt(II) | 20·2* | 0·005 | 0·013 | 0·014 | 0·015 | 0·0075 | 2·00 |
| Adriamycin | 1·32 | 0·011 | 0·054 | 0·061 | 0·07 | 0·059 | 1·36 |
| Chlorambucil | 0·58 | 0·048 | 0·20 | 0·17 | 0·03 | 0·58 | 0·91 |
| Thio TEPA | 0·42 | 0·34 | 0·15 | 0·09 | 0·08 | 0·38 | 0·77 |
| Vinblastine | 0·32 | 0·12 | 0·32 | 0·60 | 0·75 | 0·09 | 0·56 |
| Methotrexate | 3·2 | 0·48 | 0·095 | 0·31 | 0·42 | 0·65 | 0·48 |
| Average $-\log_{10}$ survival | | 1·54 | 1·16 | 1·19 | 1·00 | 1·19 | |

* Higher than the highest concentration used; SF values obtained by extrapolation.

Values for these levels and the sources of the data are given in Table II of our previous paper (Bateman *et al.*, 1979) and in Tables II and III above. They are also indicated in Figs 1–4. MeCCNU is the only drug that was not used in the previous work; on the basis of the report by Sponzo *et al.* (1973) Level A was calculated to be 2·8 μg/ml (the peak concentration between 2·5–3·5 h after administration) and Level B at 97 μg/ml.

We have read off from each dose–response curve the surviving fraction that corresponds to Level A and Level B, and the results are given in Tables II and III.

Analysis of variance was performed using the logarithm of surviving fraction as the response variable. At Level A there was no significant difference between the tumours ($P > 0{\cdot}05$) but the differences between drugs were highly significant ($P < 0{\cdot}01$). Newman and Keul's method was then used to test for difference between the drug means. The average log-survival values for the drugs are plotted

FIG. 5.—The ranking of the 8 drugs in terms of the (negative) log surviving fraction averaged over all 5 tumour lines: ○ at Level A (Table II); △ at Level B (Table III).

in Fig. 5, where it can be seen that at this level Melphalan, MeCCNU and cis-Pt were considerably more effective than the other agents. Differences amongst these 3 drugs were not significant, nor were differences amongst the other 5 drugs. However, any of the 3 most effective agents was significantly more effective than any of the 5 ($P < 0.01$).

When this analysis was repeated at Level B it was again found that the tumours were not significantly different. The apparent change in the ranking of the 3 most effective drugs was not significant, but the relative increase in effectiveness of adriamycin made it significantly more effective than the 2 lowest-ranking agents. The 3 top-ranking agents were again significantly more effective than any of the 4 lowest-ranking drugs.

Since in Tables II and III there is only one value for the response of each tumour to each drug it is not possible to use these data to comment on differences in spectrum of drug response amongst the tumour lines. We therefore selected the 4 most effective drugs and read off from Figs 1–4 the individual surviving fractions (SF) that were recorded at a drug level close to Level A: *i.e.* at 1·0 $\mu$g/ml for melphalan, 3·0 $\mu$g/ml for MeCCNU, 2–3 $\mu$g/ml for cis-Pt, and 0·3 $\mu$g/ml for adriamycin. This yielded between 1 and 4 SF values for each of the tumour–drug combinations, and these were assumed to be independent. Analysis of variance was repeated on these data, looking for interactions in tumour response. It was found that the differences amongst the 4 drugs and amongst the 5 tumour lines were both significant ($P < 0.01$) as was the interaction ($P < 0.01$). Tests showed no evidence for non-normality in the data. We may therefore conclude that in response to the 4 most effective drugs there was evidence not only for differences in responsiveness amongst the tumour lines but also for differences in their spectrum of response to the drugs.

The growth rate of the 5 melanoma lines was very similar *in vivo*, each taking about 4 weeks to reach an 8mm leg diameter. Colonies could be scored after the same culture period *in vitro* (4 weeks) and the colony growth rate was therefore also uniform. The main biological difference found amongst the lines was in melanin content. HX41 was highly melanotic, HX34, 47 and 52 were moderately melanotic, and HX50 was macroscopically amelanotic, though positive for Dopa-oxidase activity.

*Comparison of* in vitro *and* in vivo *drug sensitivity*

Studies of cell survival after *in vivo* treatment were performed and completed before the investigations of *in vitro* sensitivity began. They were, therefore, performed on earlier passages of the xenografts. Values of surviving fraction were then read off from the survival curves at the $LD_{10}$ dose levels, with extrapolation in the case of HX34 treated with MeCCNU.

The *in vivo* work used 4 drugs: mel-

TABLE IV.—*Comparison of* in vivo *and* in vitro *surviving fractions*

| Drug | HX tumour line | *In vitro* SF (at Level A) | *In vitro* SF (at Level B) | *In vivo* SF (at $LD_{10}$)* | Tumour growth delay† (at $\sim LD_{10}$) |
|---|---|---|---|---|---|
| Melphalan | 34 | 0·008 | 0·0038 | 0·0065 | NT |
| | 41 | 0·043 | 0·030 | 0·13 | 2·5 |
| | 47 | 0·015 | 0·0054 | 0·0082 | NT |
| MeCCNU | 34 | 0·027 | 0·0022 | $10^{-6}$ | NT |
| | 41 | 0·25 | 0·026 | 0·35 | 1·0 |
| | 47 | 0·041 | 0·013 | 0·004 | 5·0 |
| Adriamycin | 34 | 0·028 | 0·011 | 0·65 | NT |
| | 41 | 0·63 | 0·054 | 1·0 | 1·5 |
| | 47 | 0·42 | 0·061 | 0·86 | 0 |

* $LD_{10}$ values: melphalan, 14 mg/kg; MeCCNU, 25 mg/kg; adriamycin, 9 mg/kg.

† $\frac{\text{Treated time to double—control time to double}}{\text{Control time to double}}$.

NT = Not tested.

FIG. 6.—The correlation between the surviving fraction *in vivo* at $LD_{10}$ doses and *in vitro* at Level B concentrations. Numbers refer to the melanoma lines: HX34, 41, 47, and points corresponding to each drug are joined.

phalan, MeCCNU, adriamycin, and DTIC. Of these, DTIC could not be used *in vitro* because of its need for activation. *In vivo* data on 5 melanoma xenografts were available, but only 3 gave a satisfactory PE *in vitro*. Two more recent melanoma xenografts (HX50, 52) were therefore used in the *in vitro* studies, although *in vivo* data were unavailable for them. As a result of these constraints, the *in vivo–in vitro* comparison reduces to 3 drugs (melphalan, MeCCNU and adriamycin) and to 3 tumour lines (HX34, 41 and 47). The data are given in Table IV. The correlations of *in vivo* and *in vitro* sensitivities were slightly better at Level B than at Level A, and are illustrated in Fig. 6. For each of the 3 drugs there is a positive correlation between *in vitro* and *in vivo* sensitivity, and for MeCCNU this is statistically significant. However, in the case of adriamycin the *in vivo* responses were so small (SF often statistically indistinguishable from 1·0) that the apparent positive correlation is probably fortuitous; the only point of significance may be that HX34 gave the greatest cell kill for adriamycin *in vivo* and stood out as the most sensitive tumour *in vitro* (Fig. 3).

The broken line in Fig. 6 indicates the condition where the surviving fraction *in vivo* equals that *in vitro*. The melphalan data lie fairly close to this line. For MeCCNU 2 of the tumours gave points well away from the line, one high, one low. For adriamycin we conclude that the drug was effective *in vitro* but ineffective *in vivo*.

Some studies of *in situ* tumour-growth delay were performed by exposing mice bearing ~0·2 $cm^3$ tumours to a single $LD_{10}$ dose. Growth curves for treated and control animals were constructed from caliper measurements, and the median time taken for the tumours to double in volume was calculated. When the growth delay was calculated in multiples of the time to double of the controls, it was found that the results were mostly consistent with the cell-survival studies (Table IV): HX47 treated with MeCCNU showed the greatest delay (5·0), compared with 1·0 for HX41 with MeCCNU. In HX41 melphalan showed as expected a greater growth delay (2·5) than MeCCNU. Adriamycin gave no growth delay for HX47, but its delay in HX41 (1·5) was longer than would have been expected on the basis of cell survival. We conclude that although the growth-delay studies were not sufficiently comprehensive to allow us to draw precise conclusions, there was a hint of a positive correlation with *in vitro* chemosensitivity in the ranking of MeCCNU and melphalan: HX47 (MeCCNU) > HX41 (melphalan) > HX41 (MeCCNU).

### DISCUSSION

The data presented here are interesting from 2 points of view: in demonstrating that in a small group of xenograft lines an

*in vitro* chemosensitivity test appeared to give some indication of their *in vivo* response, and in providing new evidence on the spectrum of drug sensitivity of individual human tumours.

The decision to base the ranking of the drug sensitivities *in vitro* on estimates of drug levels achieved in man (rather than in the mouse) was because of the availability of reliable plasma-clearance data in man and the fact that correlation with tumour response in man is the ultimate objective. This was a difficult choice, and it complicates the interpretation of the data shown in Table IV and Fig. 6. It may well be, however, that differences in plasma levels between man and mouse have less effect on the results than other factors that limit the ability of an *in vitro* test to reflect *in vivo* tumour response. Drug access into the tumours is one such factor. A study of the access of $^{14}C$-melphalan into pancreatic carcinoma xenografts (HX32) showed that 60 min after injection the concentration of $^{14}C$ in the tumour reached that in the blood (Selby, *et al.*, unpublished). No doubt some of this radioactivity was by then attached to metabolites of melphalan, but nevertheless the implication is that drug access was good. In contrast, we found in mice bearing the HX34 tumour that 18 h after an $LD_{10}$ dose of adriamycin the level in liver was 0·9 $\mu$g/g but the level in the tumour was undetectable (confirming Siemann & Sutherland, 1979). This result would support the view that the lack of *in vivo* effectiveness of adriamycin was partly attributable to poor drug access into solid tumours. The insensitivity of cells exposed in diffusion chambers might also be attributable to poor drug access. In further investigations we intend to evaluate *in vitro* sensitivity at drug levels achievable in tumours.

In parallel with the *in vivo* results reported here, studies were made of the survival of melanoma xenograft cells exposed to drugs within Millipore diffusion chambers (Selby, in preparation). The chambers were removed from the treated mouse to a pre-irradiated recipient 18 h after treatment, and colonies were scored at 21 days. This "agar diffusion chamber (ADC) exposure" assay has the advantage that since drug treatment is in the mouse it is possible to use drugs that require metabolic activation. In the present work the *in vivo* and ADC exposure assays agreed reasonably well. Surprisingly, the ADC exposure results did not correlate better than the *in vivo* assay, with the *in vitro* assay.

The concept that human tumours are to some extent individual in their responsiveness to cytotoxic drugs is an important one. At present cancer chemotherapy is based on the classification of tumours by histopathological and staging criteria, each group of diseases then being treated by those drugs or drug combinations that are thought to be most effective. Clinical trials are designed to identify for each group the cytotoxic agents that give the best *average* result. It is a matter of common clinical experience that drugs which seem effective in one patient may not be so for another, but direct evidence for this has been difficult to obtain. The development of xenografts allows tissue taken from a particular patient to be tested in response to a wide range of drugs, each drug being applied to previously untreated cells. No doubt the tumour cells undergo changes (for instance in growth kinetics) when transplanted from man to mouse, but it would be surprising if these changes generated differences in spectrum of drug response between tumours that in their respective patients had similar drug sensitivities. Xenografts can therefore be a very useful experimental system in which to test this hypothesis.

Some evidence for the individuality of chemosensitivity among human tumour xenografts came from the work of Nowak *et al.* (1978). In a study of 10 xenograft lines of colorectal carcinoma treated with each of 8 chemotherapeutic agents, it was found that the responses in terms of *in situ* growth delay were on the whole poor, but that some tumour lines did relatively well with some drugs. Even actinomycin D and methotrexate, which ranked poorest overall, were the top-ranking drugs with one particular line. Individuality in response of colonic carcinoma xenografts was also seen by Osieka *et al.* (1977) and by Houghton & Houghton (1978).

The present work has provided new evidence of this phenomenon. When the analysis of variance in tumour response was restricted to the 4 most effective agents (melphalan, MeCCNU, cis-Pt and adriamycin) there was significant evidence for "interaction". The implication is that each tumour line was showing some individuality in response to these drugs.

No direct comparisons between the results of the laboratory tests and the response of the same tumours in the patient are possible. However, the overall ranking of drugs may be in line with clinical experience. MeCCNU is one of the most effective drugs in the clinical treatment of melanoma (Constanza *et al.*, 1977) and melphalan, used in high doses, is proving moderately effective (McElwain *et al.*, 1979, and in preparation). Cis-Pt has not been widely used. In keeping with the *in vivo* survival data, adriamycin is inactive in clinical melanoma (Sieper *et al.*, 1975).

The translation of information on chemosensitivity from a short-term laboratory test to clinical practice must take into account the drug schedules that are in clinical use. Where infrequent large doses are used the results of an *in vitro* test could give valuable information. With drugs such as methotrexate that are given by infusion or in protracted schedules the test will be less useful, and may underestimate the clinical effectiveness of a drug. The other major difficulty with *in vitro* chemosensitivity tests is the danger of wrongly evaluating drugs that are metabolised *in vivo* and thereby inactivated or transformed into metabolites that are more active than the parent drug. Our use of the "ADC exposure" assay is intended to alleviate this problem, and the detailed results will be described elsewhere. With experience, we would expect to learn that certain drugs are over- or under-rated by an *in vitro* test, and modify accordingly the concentration at which sensitivity should be evaluated.

The present work is regarded as a modest step towards the validation and development of *in vitro* chemosensitivity tests for human tumours. The results are encouraging, but they demonstrate the need for more detailed pharmacokinetic studies and for direct comparisons of the laboratory tests with the response of patients.

We are grateful for the support and encouragement of Professor M. J. Peckham, for helpful discussions with Mrs V. D. Courtenay, who developed the *in vitro* cloning assay, and for statistical advice from Miss M. Jones of the Division of Epidemiology. The work on tumour growth delay was performed by Mr J. Gibbs. The project was partly supported by NCI Grant Number NO1-CA20519.

### REFERENCES

BATEMAN, A. E., PECKHAM, M. J. & STEEL, G. G. (1979) Assays of drug sensitivity for cells from human tumours: *In vitro* and *in vivo* tests on a xenografted tumour. *Br. J. Cancer*, **40**, 81.

CONSTANZA, M. E., NATHANSON, L., SCHOENFIELD, D. & 5 others (1977) Results with methyl-CCNU and DTIC in metastatic melanoma. *Cancer*, **40**, 1010.

COURTENAY, V. D. & MILLS, J. (1978) An *in vitro* colony assay for human tumours grown in immune-suppressed mice and treated *in vivo* with cytotoxic agents. *Br. J. Cancer*, **37**, 261.

HOUGHTON, P. J. & HOUGHTON, J. A. (1978) Evaluation of single-agent therapy in human colorectal tumour xenografts. *Br. J. Cancer*, **37**, 833.

MCELWAIN, T. J., HEDLEY, D. W., GORDON, M. Y., JARMAN, M., MILLAR, J. L. & PRITCHARD, J. (1979) High dose melphalan and non-cryopreserved autologous bone marrow-treatment of malignant melanoma and neuroblastoma. *Exp. Haematol.*, **7**, *Suppl.* 5, 360.

NOWAK, K., PECKHAM, M. J. & STEEL, G. G. (1978) Variation in response of xenografts of colorectal carcinoma to chemotherapy. *Br. J. Cancer*, **37**, 576.

OSIEKA, R., HOUCHENS, D. P., GOLDIN, A. & JOHNSON, R. K. (1977) Chemotherapy of human colon cancer xenografts in athymic nude mice. *Cancer*, **40**, 2640.

SELBY, P. J., THOMAS, J. M., MONAGHAN, P., SLOANE, J. & PECKHAM, M. J. (1980) Human tumour xenografts established and serially transplanted in mice immunologically deprived by thymectomy, cytosine arabinoside and whole-body irradiation. *Br. J. Cancer*, **41**, 52.

SIEMANN, D. W. & SUTHERLAND, R. M. (1979) Pharmacokinetics of adriamycin in normal and neoplastic tissue following single and multiple drug doses. *Int. J. Radiat. Oncol.* (in press).

SIEPER, W. J., MASTRANGELO, M. J. & BELLET,

R. E. (1975) Phase II study of adriamycin in patients with metastatic melanoma. *Cancer Chemother. Rep.*, **59**, 1181.

SMITH, I. E., COURTENAY, V. D. & GORDON, M. Y. (1976) A colony forming assay for human tumour xenografts using agar in diffusion chambers *Br. J. Cancer*, **34**, 476.

SPONZO, R. W., DEVITA, V. T. & OLIVIERO, V. T. (1973) Physiological disposition of 1-(2-chloroethyl)-3-cyclohexyl-1-nitrosourea (CCNU) and 1-(2-chloroethyl)-3-(4-methylcyclohexyl)-1-nitrosourea (MeCCNU) in man. *Cancer*, **31**, 1154–1156.

STEEL, G. G., COURTENAY, V. D. & ROSTOM, A. Y. (1978) Improved immune-suppression techniques for the xenografting of human tumours. *Br. J. Cancer*, **37**, 224.